# Optimization of Hybrid PEO/P(L/G/TMC) Coatings on WE43B Magnesium Alloy: Effect of Polymer Layer Number on Surface Properties

**DOI:** 10.3390/ma19091688

**Published:** 2026-04-22

**Authors:** Barbara Rynkus, Ada Orłowska, Karolina Wilk, Joanna Jaworska, Katarzyna Nowińska, Karolina Szawiraacz, Justyna Więcek-Chmielarz, Krzysztof Lukaszkowicz, Mariusz Sandomierski, Piotr Kałużyński, Maciej Sowa, Janusz Szewczenko

**Affiliations:** 1 Department of Biomaterials and Medical Device Engineering, Faculty of Biomedical Engineering, Silesian University of Technology, Roosevelta 40, 41-800 Zabrze, Poland; ada.orlowska@polsl.pl (A.O.); karolina.wilk@polsl.pl (K.W.); 2 Centre of Polymer and Carbon Materials, Polish Academy of Sciences, M. Curie-Skłodowskiej 34, 41-819 Zabrze, Poland; jjaworska@cmpw-pan.pl; 3 Department of Applied Geology, Faculty of Mining, Safety Engineering and Industrial Automation, Silesian University of Technology, Akademicka 2, 44-100 Gliwice, Poland; katarzyna.nowinska@polsl.pl; 4 Institute of Metallurgy and Materials Science, Polish Academy of Sciences, Reymonta 25, 30-059 Kraków, Poland; szawiraacz.k@imim.pl (K.S.); wiecek.j@imim.pl (J.W.-C.); 5 Department of Engineering Materials and Biomaterials, Faculty of Mechanical Engineering, Silesian University of Technology, Konarskiego 18A, 44-100 Gliwice, Poland; krzysztof.lukaszkowicz@polsl.pl; 6 Institute of Chemical Technology and Engineering, Poznan University of Technology, Berdychowo 4, 61-131 Poznań, Poland; mariusz.sandomierski@put.poznan.pl; 7 Department of Optoelectronics, Faculty of Electrical Engineering, Silesian University of Technology, B. Krzywoustego 2, 44-100 Gliwice, Poland; piotr.kaluzynski@polsl.pl; 8 Department of Inorganic Chemistry, Analytical Chemistry and Electrochemistry, Faculty of Chemistry, Silesian University of Technology, B. Krzywoustego 6, 44-100 Gliwice, Poland; maciej.sowa@polsl.pl

**Keywords:** magnesium alloy, plasma electrolytic oxidation, P(L/G/TMC), polymer coating, hybrid coatings

## Abstract

Magnesium alloys are promising materials for orthopedic applications due to their biodegradability and mechanical properties compatible with bone. However, their rapid degradation in physiological environments limits clinical use. In this study, WE43B magnesium alloy was coated with a PEO layer followed by a P(L/G/TMC) polymer applied via ultrasonic spraying. The influence of polymer layer number (10, 20, 30) on coating properties was systematically investigated. Scanning electron microscopy (SEM) analysis revealed an approximately fourfold reduction in porosity after polymer deposition, with progressive pore filling at higher layer numbers, while Fourier transform infrared spectroscopy (FT-IR) mapping indicated uniform polymer coverage. Compared to PEO alone, polymer-modified samples exhibited an approximately 7-fold increase in water contact angle, a ~50% reduction in surface roughness, and improved adhesion. Degradation-related analyses, including ion release, post-immersion SEM, and scanning acoustic microscopy (SAM), indicated that increasing polymer thickness effectively limited degradation processes. Ion release decreased by ~40–50% for the 30-layer coating compared to PEO, with the most pronounced reduction observed between the uncoated PEO and polymer-modified samples. These results demonstrate that the number of polymer layers plays a key role in controlling the barrier properties and stability of hybrid PEO/polymer coatings under simulated physiological conditions.

## 1. Introduction

Magnesium alloys have become one of the most promising groups of new-generation biomaterials used in the production of temporary orthopedic implants due to their unique combination of biodegradability, biocompatibility and mechanical properties [1,2,3]. Unlike conventional metal implants, such as titanium and its alloys or stainless steel, which remain in the body permanently, magnesium-based implants gradually degrade in the physiological environment and are resorbed by the body. This feature eliminates the need for additional surgery, thereby reducing the risk of post-operative complications, infections and patient discomfort, as well as lowering the overall cost of treatment [4,5]. In addition, magnesium has a low density and a modulus of elasticity that is closest to that of human bone among other metallic biomaterials, which minimizes the risk of stress shielding, which negatively affects the fracture healing process [6,7]. In addition to their favorable mechanical properties [8,9], magnesium alloys also have excellent biological properties [10,11]. Degradation products, mainly magnesium ions, occur naturally in the human body and participate in many physiological processes, including bone mineralization. Controlled release of Mg^2+^ ions can therefore stimulate osteogenic activity and accelerate bone regeneration [12,13].

However, despite the many advantages associated with the use of magnesium-based alloys, the main obstacle to their widespread clinical application remains poor corrosion resistance and excessive degradation in the physiological environment [14]. Uncontrolled corrosion leads to premature loss of mechanical integrity, local alkalization and hydrogen release, which can interfere with tissue healing [15,16,17,18]. Therefore, current research focuses on adjusting the surface properties of magnesium alloys to achieve controlled degradation that matches the rate of bone healing, ensuring both structural stability and a favorable biological response. One of the most effective ways to achieve this balance is to develop appropriate surface modifications in the form of protective surface coatings to control corrosion kinetics while maintaining biofunctionality.

Many surface modification strategies have been investigated to improve the protective and functional properties of magnesium alloys. Among them, plasma electrolytic oxidation (PEO) stands out as an effective surface treatment method, which creates a hard, ceramic layer that effectively improves the corrosion resistance of the magnesium substrate but also ensures wear resistance and biocompatibility [19,20,21]. The PEO process allows for the production of a wide range of functional coatings thanks to the possibility of modifying the electrolyte composition with appropriately selected additives [22,23,24]. The selection of specific compounds allows for the targeted shaping of the properties of the layer produced on the substrate. For example, the introduction of calcium and phosphorus ions into the electrolytic solution promotes the formation of bioactive coatings with increased osseointegration potential [25,26,27]. However, a typical PEO coating is porous and often covered with microcracks formed during the formation of the layer, which allows electrolytes to penetrate the substrate, causing local corrosion and delamination during prolonged exposure to physiological solutions [28]. Therefore, PEO coatings alone are often insufficient to provide the long-term stability required for biodegradable implants.

The application of an additional polymer coating on magnesium alloys after the plasma electrolytic oxidation (PEO) process is currently one of the most promising strategies for modifying the surface of degradable biomaterials. The primary function of such a coating is to seal and protect the substrate while maintaining the bioactivity and beneficial biological properties of the surface [29,30,31]. Applying a polymer to the PEO coating significantly reduces its open porosity, which translates into a decrease in the diffusion rate of chloride ions and a delay in the initiation of corrosion processes. The polymer layer acts as an additional physical barrier that reduces the contact of the aggressive physiological environment with the metal substrate, allowing for a more gradual and controlled degradation of the implant. The use of a polymer layer therefore makes it possible to create a hybrid system that acts as a regulator of the corrosion rate, helping to achieve a degradation profile that is tailored to the healing and tissue remodelling process.

One of the most common approaches to sealing the pores of PEO (also known as microarc oxidation—MAO) coatings is the use of biodegradable polymers. Li et al. [32] demonstrated that MAO with poly(lactic-co-glycolic acid) (PLGA) coating significantly reduces the rate of magnesium alloy degradation in both in vitro and in vivo studies, while promoting bone tissue formation around the implant. However, the authors noted that polymer degradation leads to local damage to the coating and intense local corrosion after the protective layer is lost.

Similar conclusions were obtained in studies on MAO with poly(l-lactic acid) (PLLA) [33] and MAO with polycaprolactone (PCL) [34] hybrid coatings on magnesium alloys. In both cases, the authors applied an additional polymer layer to the porous MAO coating in order to reduce the rate of degradation and improve the biocompatibility of the implant material. It was demonstrated that both PLLA and PCL effectively penetrate the micropores of the MAO layer, leading to a significant reduction in the rate of magnesium degradation and an improvement in biological response compared to the MAO coating alone. At the same time, the authors observed that long-term polymer degradation leads to local loss of coating continuity and the development of local corrosion, which can rapidly accelerate substrate degradation. In vitro and in vivo studies have shown improved adhesion, proliferation and differentiation of bone cells, as well as reduced inflammatory response, confirming the beneficial effect of the polymer layer on osteogenesis.

In response to these limitations, increasing attention is being paid to degradable polymers, in particular poly(trimethylene carbonate) (PTMC). Ghanbari et al. [29] proposed a multilayer PEO/PTMC system in which the flexible polymer effectively filled the pores of the PEO coating, leading to a significant improvement in corrosion resistance and a reduction in pH changes during long-term incubation in SBF. Importantly, thanks to the neutral degradation products of PTMC, the chemical stability of the environment around the implant was maintained, and additional functionalization of the surface with polydopamine enabled intensive apatite mineralization and improved bone cell adhesion and proliferation. Qian et al. [35] developed a new hybrid MAO/PTMC coating system on AZ31 magnesium alloy, which aimed to extend the durability of the material while maintaining biocompatibility. They demonstrated that the MAO coating with an additional PTMC polymer layer reduces the corrosion rate by up to 851 times compared to the substrate, achieving 99.9% protection efficiency. During 21 days of incubation in solution, significantly reduced pH changes and reduced Mg^2+^ ion release were observed, with no pitting corrosion. The authors emphasised that the micro- and nanoporous structure of the MAO layer promotes strong anchoring of PTMC and can act as a carrier for drugs or bioactive substances, making the MAO/PTMC system a promising solution for bone implants with controlled biodegradation.

The results of previous studies clearly indicate that combining a porous PEO/MAO layer with a suitably selected polymer coating allows for effective control of the magnesium degradation process and shaping of the biological response to the implant. At the same time, the literature shows that the protective and biofunctional properties of such systems are strongly dependent on the nature of the polymer, its degradation mechanism and the degree of sealing of the PEO structure. Issues related to the architecture of the polymer coating, including the influence of the number and thickness of the applied layers on ion transport and long-term barrier properties, remain insufficiently understood.

The aim of this study was to develop and optimize hybrid PEO/P(L/G/TMC) coatings applied to WE43B magnesium alloy in order to ensure controlled material degradation.

WE-series magnesium alloys are increasingly recognized as promising candidates for biomedical applications. While most studies focus on pure magnesium or AZ-series alloys, WE-type alloys offer superior mechanical properties and improved corrosion resistance due to the presence of rare-earth elements, without incorporating potentially harmful elements such as aluminum [36,37]. Previous studies have demonstrated good biocompatibility of WE43-based alloys, and notably, similar compositions have already been used in clinically tested and commercially available biodegradable implants [38,39]. Therefore, WE43B represents a relevant and forward-looking model material for the development of advanced surface modifications for next-generation biodegradable implants.

P(L/TMC) is a biocompatible homopolymer; it is frequently copolymerized with different monomers. Copolymerization is a well-known strategy used to obtain materials with moderate properties. In this study, a trimethylene carbonate-based terpolymer was used, consisting of three different kinds of structural units. By introducing lactide and glycolide into P(L/TMC), modification of the polymer chain microstructure was achieved, allowing for tailoring of material properties, such as improved degradation rates.

The microstructure of poly(lactide-glycolide-trimethylene carbonate) obtained by ring-opening polymerization has been characterized in detail by Gębarowska et al. [40]. Its specific chain microstructure can ensure a favorable drug delivery profile [41] as well as good mechanical properties [42]. Thus, P(L/G/TMC) was selected as a coating material for the WE43B magnesium alloy.

The main objective of the hybrid modification is to slow down the degradation process of the magnesium alloy so that it retains its mechanical properties for the period necessary for fracture stabilization and proper bone healing. Therefore, this paper presents a systematic assessment of the effect of the number of polymer layers on the surface properties and performance of the hybrid system. The work focuses on understanding the behavior of the P(L/G/TMC) polymer in combination with a pre-formed PEO layer, with particular emphasis on how a controlled, layer-by-layer deposition strategy influences morphology, physical properties, and barrier performance.

While hybrid PEO/polymer coatings have been widely reported, the novelty of this study lies primarily in the processing strategy and precise control of the polymer layer architecture. In particular, the use of ultrasonic spraying, unlike commonly used dip-coating techniques, allows for the controlled and repeatable application of thin layers and more uniform coverage of complex structures [43,44,45].

To comprehensively evaluate the properties of the developed coatings, a range of complementary characterization techniques was employed. Surface morphology was analyzed using scanning electron microscopy (SEM), while Fourier transform infrared spectroscopy (FT-IR) was used to confirm the presence and uniformity of the polymer layer. Surface topography was assessed by optical profilometry, and wettability along with surface free energy (SFE) measurements were performed to characterize interfacial properties. The adhesion of the coatings to the substrate was evaluated using scratch testing. Degradation behavior was investigated through a 28-day immersion test in phosphate-buffered saline (PBS), including ion release analysis and post-immersion SEM observations to assess surface changes. Furthermore, scanning acoustic microscopy was applied to identify regions susceptible to hydrogen evolution and to compare the structural integrity of the coatings before and after immersion.

This approach allows for a direct correlation between the number of applied layers and the resulting functional properties, providing a more detailed insight into structure-property relationships in hybrid coatings. Consequently, the study offers practical guidelines for the design of hybrid polymer/PEO systems with tailored degradation behavior.

As a result, the work contributes to the field of biodegradable magnesium-based biomaterials by demonstrating that, beyond material selection, the processing route and precise control over coating architecture play a critical role in determining the performance and stability of hybrid protective systems in physiological conditions.

## 2. Materials and Methods

### 2.1. Specimen Preparation

Magnesium alloy rod WE43B (Mg-4Y-3Nd) (Goodfellow, Hamburg, Germany) was cut to obtain samples as discs of 11 mm diameter and 4 mm thickness. The magnesium alloy samples were ground with SiC waterproof sandpaper to a gradation of #1200. The samples were then washed with isopropanol on an ultrasonic cleaner for 20 min. The degreased samples were etched with a 1% nitric acid solution for 30 s. Finally, the samples were washed with demineralized water and dried with a stream of cool air.

The plasma electrolytic oxidation (PEO) treatment of the WE43B magnesium alloy was carried out according to the procedure described in detail in our previous study [46]. In brief, coatings were produced in a two-step process, incorporating both anodic and cathodic polarizations, using an AC+DC high-voltage power supply. In the first stage, samples were oxidized in a phosphate-based electrolyte containing 12 g/L sodium hexametaphosphate (NaPO_3_)_6_ and 0.05 M KOH until a voltage of 350 V was reached. In the second step, oxidation was continued for 10 min in a modified electrolyte based on the previous solution with the addition of 1.038 g/L calcium acetate [Ca(CH_3_COO)_2_·H_2_O]. This allowed to set the Ca:P ratio in the solution to 1:20. The process was performed under 10 °C with continuous stirring, with a duty ratio (DR) of 50% and a current ratio (R = cathodic current/anodic current) of 1.6.

### 2.2. Polymer Coating Application

The terpolymer P(L/G/TMC) was synthesized via a ring-opening polymerization of three monomers: glycolide (12.76 g), L-lactide (110.88 g), and trimethylene carbonate (TMC, 22.44 g). The reaction was carried out in the molten state at 120 °C under an argon atmosphere. Zirconium(IV) acetylacetonate, Zr(acac)_4_ (0.357 g), was used as an initiator, with a molar ratio of initiator to total monomers of 1:1500. Polymerization was conducted in a single step by melting all monomers simultaneously, followed by the addition of the initiator. The mixture was continuously stirred until the completion of the reaction, which lasted for 120 h.

After polymerization, the obtained terpolymer was purified by dissolution in chloroform and subsequent precipitation in cold methanol. The product was then dried under vacuum to constant weight. Finally, 1 g of the P(L/G/TMC) polymer was dissolved in 99 mL of dichloromethane to obtain a 1 wt% polymer solution, which was used for the preparation of the coating layers.

The ultrasonic spray-coating technique was selected due to its superior control over layer thickness, uniformity, and deposition precision compared to conventional coating approaches such as dip-coating or spin-coating. Owing to the fine atomization of the polymer solution generated by ultrasonic vibration, this method enables the formation of smooth, homogeneous, and defect-reduced films while minimizing solvent waste and allowing precise regulation of the deposition rate. Such control is particularly advantageous when working with porous or microstructured substrates, including PEO-treated surfaces, where traditional immersion-based processes often lead to coating delamination, uneven buildup, or incomplete pore coverage. By contrast, ultrasonic spray-coating ensures gradual and well-controlled film formation, enhancing interfacial stability and promoting uniform penetration of the polymer into the PEO layer [43,44,45].

To systematically assess how the thickness of the polymer coating influences the functional behavior of the hybrid system, three coating thickness levels, corresponding to 10, 20, and 30 deposited layers, were selected. These values were chosen to provide a stepwise increase in coating thickness while maintaining precise control over layer deposition enabled by ultrasonic spraying. This range enables evaluation of property changes with increasing layer number and assessment of whether further thickening remains beneficial. At the same time, the selected number of layers ensures a representative yet experimentally manageable dataset, enabling reliable comparison of morphology, integrity, and functional properties across the hybrid systems.

Polymer coatings were applied onto the PEO-treated WE43B substrates using an ultrasonic spray-coating system (ExactaCoat, Sono-Tek, New York, NY, USA), operating under the deposition parameters summarized in Table 1. The samples were designated as follows: PEO—reference sample after plasma electrolytic oxidation only, PEO+PC-10, PEO+PC-20, and PEO+PC-30, where PC refers to the polymer coating and the number indicates the number of deposited layers.

### 2.3. Surface Characterization

The surface morphology assessment was carried out using a scanning electron microscope (SEM) (TESCAN VEGA, Brno, Czech Republic) with SE detectors at an energy of 3–10 keV. Observations were performed at 3 and 5 keV and 1 nA in high vacuum. Image analysis of SEM micrographs (×250 magnification) of the coatings was performed using ImageJ 1.54g software (NIH, Bethesda, MD, USA) to quantitatively describe differences between surface variants. Prior to binarization, images were subjected to a despeckle filter (median filtering) to reduce noise, followed by contrast enhancement to improve grayscale distribution. Binarization was carried out using automatic thresholding based on the Huang method to distinguish pores from the oxide/polymer. Then, pore detection and analysis were performed using particle analysis, allowing for the determination of pore count and size distribution. Based on this analysis, three characteristic parameters were determined for each image: pore density (number of pores per mm^−2^), open porosity (%), and equivalent pore diameter (μm). The equivalent diameter was calculated from the mean pore area, assuming circular pore geometry, as the diameter of a circle with an equivalent area.

In addition, for a more detailed characterization of polymer coatings, the thickness of the polymer coatings was estimated using optical profilometry. For this purpose, polymer layers (10, 20, and 30) were deposited on BK7 flat glass substrates under the same process conditions as for the coated samples. The measurements were performed using a Bruker ContourX-100 optical profilometer (Bruker Corporation, Billerica, MA, USA) operating in Vertical Scanning Interferometry (VSI) mode, equipped with a Nikon 10×/0.30 DI objective and a 0.55× field-of-view lens, under white light illumination, for a scanned surface area of approximately 1480 × 1230 µm^2^. The step height between the coated and uncoated regions was measured, and the coating thickness was determined based on the ΔZ value obtained from the surface profiles.

Accurate measurement of polymer layer distribution over large surface areas is essential for reliable evaluation of surface modification effectiveness. Therefore, FT-IR mapping was employed in the present study. The spatial distribution of the polymer coating on the surface of a magnesium alloy was analyzed using a LUMOS II FT-IR microscope (Bruker, Billerica, MA, USA). Measurements were performed in reflection mode. Mapping was conducted over nearly the entire surface of the analyzed sample. The mapped area consisted of 225 measurement points arranged in a 15 × 15 grid, with each pixel covering an area of 500 × 500 µm, resulting in a total mapped area of 7.5 × 7.5 mm. Spectra were collected using a single-element TE-MCT detector (Bruker, Billerica, MA, USA). At each measurement point, 240 scans were accumulated to improve the signal-to-noise ratio. Background spectra were recorded using a gold reference prior to sample measurements. FT-IR maps were generated based on the band areas of three selected characteristic bands of the polymer. This approach enabled a comparative evaluation of the spatial distribution and uniformity of the deposited polymer layers across the sample surface.

The surface topography studies were carried out with 3D Surface Metrology Microscope Leica DCM8 (Leica Microsystems GmbH, Wetzlar, Germany) using confocal differentiation method. The resulting images were processed in Leica Map Premium 10 software (Leica Microsystems GmbH, Wetzlar, Germany). Green light and a 20× magnification were used. Measurements were carried out globally over an area of 877 × 660 μm. Five samples from each variant were measured at three spots, with three profiles created at each spot, based on which the values of Sa (arithmetic mean deviation of the profile from the mean surface), Ra (arithmetic mean deviation of the profile from the mean line) and Rz (height of roughness according to ten points of the profile) were determined.

The surface wettability test was performed using an Attension Theta Flex (Biolin Scientific, Gothenburg, Sweden) optical tensiometer. The contact angle Θ was determined using the sessile drop method, using distilled water (H_2_O) and diiodomethane (I_2_CH_2_) as the mediums in volumes of 1.5 mm^3^ for each of the droplets. The measurement duration was 60 s with a sampling frequency of 1 Hz. The surface free energy (SFE) was determined from the average contact angle values of H_2_O and I_2_CH_2_ using the Owens, Wendt, Rabel, and Kaelble (OWRK) method. Measurements were taken at three spots for three samples per variant.

Adhesion of the coatings to the substrate was evaluated by the scratch-test method using an RST^3^ Revetest (Anton Paar GmbH, Graz, Austria). Tests were conducted with a Rockwell penetrator, and the loading force was built up from 1 N to 30 N. The scratch length was 3 mm, and the sliding speed of the table was ~10 mm/min. The values of the critical load causing layer breakage were determined from microscopic observations and frictional forces. Three measurements were taken on each sample.

### 2.4. Immersion Test

The analysis of metal ion release into PBS solution from the surface of the tested samples was performed using inductively coupled plasma atomic emission spectrometry (ICP-AES) with JY 2000 spectrometer (Horiba Jobin Yvon, Paris, France). The concentrations of Mg, Y, Nd, Ca and P ions from the samples were determined. Reference curves were developed for Merck reference materials. PBS solutions with a volume of 50 mL were tested after incubating the samples for 28 days at 37 °C. The results obtained were presented as the density of ions released per unit area using the following conversion (1). Five samples from each variant were tested.

(1) $$d=\left(\frac{ppm\times V}{s}\right)\times 1000,$$

where:

d—density of ions released from the surface [μg/cm^2^],

ppm—number of ions in the solution,

V—volume of the solution,

s—surface area of the sample.

Following removal from the PBS solution, the samples were left to dry at ambient temperature, after which the surface morphology post-degradation was examined via scanning electron microscopy (SEM). 

Scanning acoustic microscopy (SAM) tests were conducted to qualitatively assess the behavior of protective coatings on magnesium alloy in an aquatic environment, with particular emphasis on local coating stability and the identification of areas susceptible to degradation and the initiation of corrosion processes. Samples with a hybrid PEO+PC coating in their initial state and after a 28-day immersion test were analyzed in an aqueous environment, which acted as a coupling medium for ultrasonic waves.

**Figure 1:**
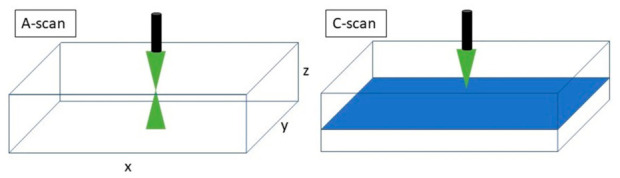
Scanning patterns in scanning acoustic microscopy (SAM).

Samples were examined using a scanning acoustic microscope (SAM, EVOLUTION II, KSI, Herborn, Germany) operating in pulse-echo reflection mode. The system was equipped with a focused piezoelectric transducer operating at a central frequency of 75 MHz. The transducer consisted of a piezoceramic layer deposited on a sapphire substrate coated with a ZnO layer, enabling the generation and detection of high-frequency ultrasonic pulses with high penetration capability. During measurements, the transducer was immersed in redistilled water, which served as an acoustic coupling medium between the transducer and the sample surface. Ultrasonic pulses generated by the piezoelectric element were transmitted into the sample, and the reflected acoustic signals returning from the sample interfaces were received by the same transducer. The reflected signals were converted into electrical signals and digitally processed to generate acoustic images displayed as pixel-based maps. Measurements were performed using the C-scan imaging mode, which represents a compilation of multiple A-scan signals acquired along the XY scanning plane at a selected focal depth within the sample (Figure 1). In this mode, the amplitude of the reflected echo was recorded at each scanning position, allowing visualization of surface features and subsurface inhomogeneities within a defined focal plane. Acoustic images were analyzed to assess surface continuity, the presence of internal defects, and variations in acoustic impedance. All measurements were performed under identical experimental conditions to ensure comparability between samples.

The analysis of the SAM results was qualitative and comparative. The method was used as a tool to aid in the identification of preferential sites of coating degradation under immersion conditions, rather than as a direct method for the quantitative measurement of corrosion rates.

## 3. Results and Discussion

### 3.1. Surface Morphology

**Figure 2:**
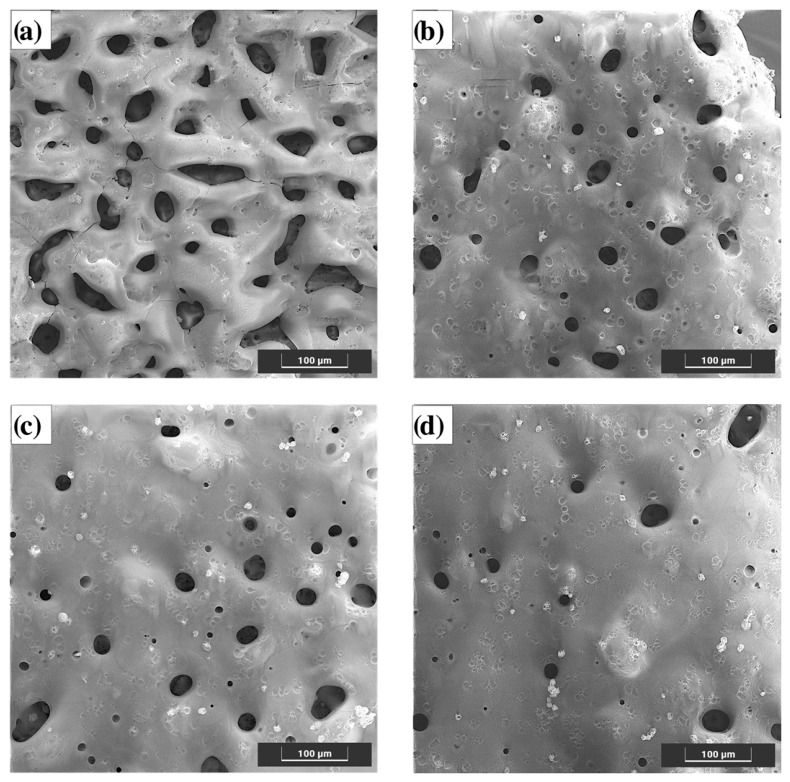
Surface morphology of the tested samples: (**a**) initial state—PEO, (**b**) PEO with 10 polymer layers, (**c**) PEO with 20 polymer layers, (**d**) PEO with 30 polymer layers.

The surface of the PEO coating (Figure 2a) exhibits the characteristic porous microstructure typical of plasma electrolytic oxidation layers. Numerous open pores of irregular size and shape can be observed, resulting from the localized microdischarge events occurring during the oxidation process [19,20,21,22,23,24,46]. Such morphology provides a favorable base for polymer infiltration but also facilitates electrolyte penetration, which may accelerate substrate degradation when exposed to physiological media.

A quantitative analysis of SEM images was performed to evaluate the effect of polymer deposition on the surface morphology of the coatings, including pore density, porosity, and equivalent pore diameter (Table 2).

The results confirm that the application of the polymer coating leads to a significant reduction in all analyzed parameters compared to the PEO layer. For the PEO+PC-10 sample (Figure 2b), pore density decreased by approximately 28% (from 210 to 151 pores/mm^2^), while porosity was reduced by about 67% (from 12.9% to 4.3%). The equivalent pore diameter also decreased by approximately 32% (from 28.0 µm to 19.0 µm), indicating partial filling of the PEO pores, which is consistent with the SEM observations.

Further increasing the number of polymer layers to 20 (Figure 2c) resulted in a continued, though less pronounced, reduction in pore density (by approx. 34% relative to PEO) and porosity (by approx. 71%), with the equivalent diameter decreasing by approximately 33%. This corresponds to a more uniform surface morphology with reduced pore size and density.

For the PEO+PC-30 sample (Figure 2d), the lowest values of all parameters were observed. Pore density decreased by approximately 42%, porosity by around 74%, and equivalent pore diameter by about 34% compared to the PEO coating. These results indicate the formation of the most compact and continuous coating, with the polymer effectively sealing the majority of the pores. However, the presence of residual small pores confirms that complete sealing of the PEO structure was not achieved.

Importantly, the aim of the polymer deposition process was not to fully seal the porous PEO layer, but rather to significantly reduce the pore size and number in order to improve coating tightness and limit rapid solution penetration. Maintaining a certain level of surface porosity is beneficial, as it allows for gradual ion exchange and controlled degradation of the underlying magnesium alloy, which are key features for bioresorbable orthopedic implants. Therefore, the hybrid coating design was optimized to balance barrier functionality and controlled biodegradability, ensuring both improved corrosion protection and preservation of the material’s biofunctional character.

These observations indicate that the polymer progressively penetrates and seals the porous oxide layer, improving its surface integrity and potential barrier performance. The observed morphological evolution suggests that increasing the number of polymer layers enhances coating continuity and smoothness, which is expected to positively influence the corrosion resistance and adhesion stability of the hybrid coating system.

### 3.2. Fourier Transform Infrared Spectroscopy (FT-IR) and Coating Thickness

To better understand the relationship between the number of deposited polymer layers and the resulting coating properties, additional thickness measurements were performed. Since the thickness of the coatings was initially defined only by the number of deposition layers, it was necessary to estimate the actual thickness of the polymer layer.

**Figure 3:**
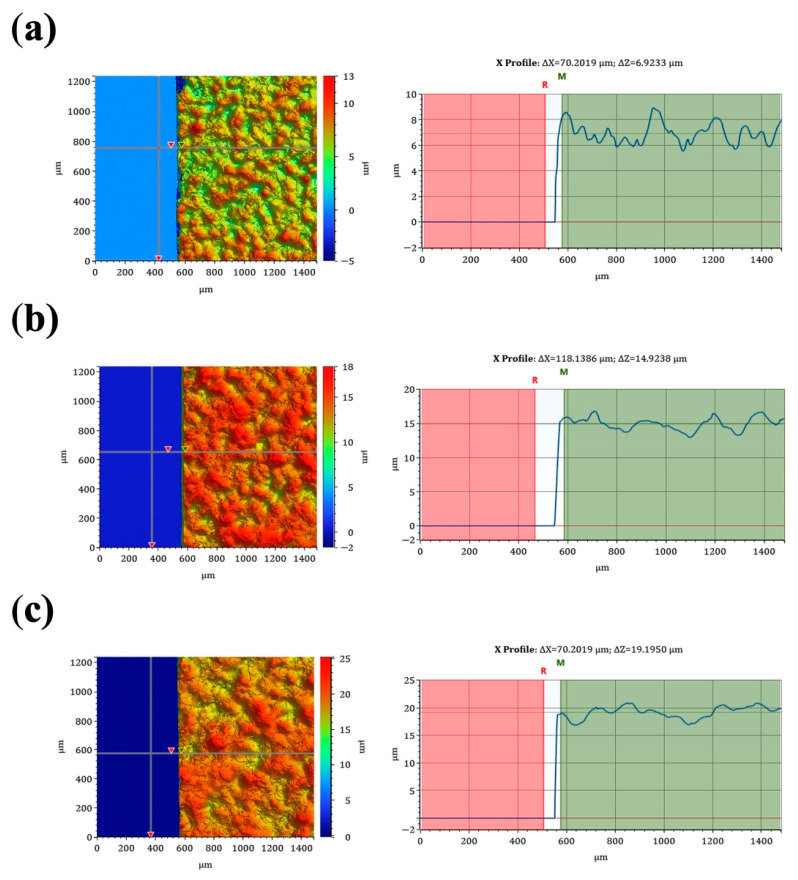
Thickness of polymer coatings determined by optical profilometry for samples with (**a**) 10, (**b**) 20, and (**c**) 30 deposited layers, based on step height (ΔZ) measurements on glass substrates.

The results confirmed that the thickness of the polymer coatings increased with the number of deposited layers (Figure 3). The measured thickness was approximately 7 µm for 10 layers, ~15 µm for 20 layers, and ~20 µm for 30 layers.

The observed increase in thickness indicates a clear dependence on the number of deposition layers, although the relationship is not strictly linear. This suggests that subsequent layers partially overlap and contribute to coating densification rather than forming entirely independent layers. Such behavior is consistent with the deposition mechanism and may contribute to the progressive improvement of barrier properties observed for coatings with a higher number of polymer layers.

FT-IR was employed as an additional technique for the characterization of the PEO coating before and after the deposition of the polymer layer. This method enabled the identification of chemical bonds present on the surface and provided insight into the spatial distribution of the polymer deposited on the PEO-treated magnesium alloy.

**Figure 4:**
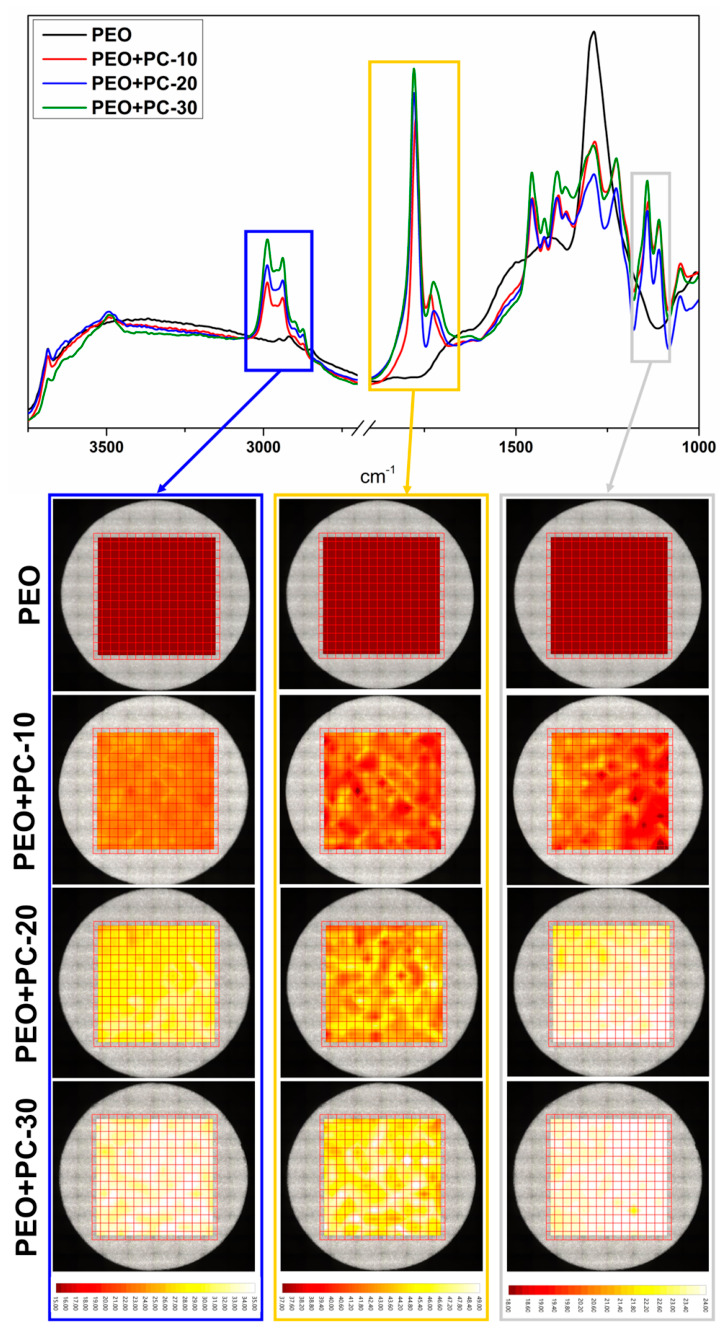
FT-IR spectra obtained in reflection mode and maps for the marked bands.

As shown in the FT-IR spectra (Figure 4), the PEO-coated sample did not exhibit characteristic absorption bands in the selected spectral ranges, indicating the absence of organic functional groups on the surface. In contrast, after the application of the polymer coating, additional absorption bands appeared, which can be attributed to the presence of the P(L/G/TMC) polymer on the surface of the alloy. This observation confirms the successful deposition of the polymer layer onto the PEO coating.

For FT-IR mapping, three spectral ranges were selected to enable a detailed analysis of the polymer distribution across the surface of all sample variants. The selected spectral ranges correspond to stretching vibrations of C–H bonds (3050–2850 cm^−1^), C=O bonds (1890–1690 cm^−1^), and C–O bonds (1180–1085 cm^−1^) characteristic of ester present in the polymer structure [47]. Importantly, no absorption bands were detected in these spectral ranges for the PEO sample, which was further confirmed by FT-IR mapping. For all three selected bands, no signal was recorded at any measurement point for the PEO coating, confirming that these bands are exclusively associated with the polymer layer.

In the case of polymer-modified samples, the FT-IR chemical maps clearly demonstrate a uniform distribution of the selected bands over the entire analyzed surface. This homogeneous signal distribution indicates that the polymer layers are evenly deposited, providing indirect confirmation of the continuity and uniformity of the coating. Furthermore, an increase in the integrated band area was observed with an increasing number of deposited polymer layers. This trend reflects the gradual buildup of the polymer coating and confirms that successive deposition cycles effectively increase the amount of polymer present on the surface.

Notably, a uniform spatial distribution was observed consistently for all three analyzed bands across all polymer-coated samples. This result confirms not only the effectiveness of the polymer deposition process but also the homogeneous coverage of the surface over the entire analyzed area. The FT-IR mapping results therefore corroborate the morphological observations and demonstrate that ultrasonic spray coating enables the formation of continuous and uniformly distributed polymer layers on porous PEO coatings.

### 3.3. Surface Topography

Table 3 summarizes the surface roughness parameters (Ra, Rz and Sa) obtained for the PEO coating and for hybrid PEO/polymer coatings with different numbers of polymer layers. Surface topography tests showed significant differences between samples subjected only to the PEO process and samples additionally modified with a polymer coating. The surface after the oxidation process was characterized by the highest values of the roughness parameters Sa, Ra and Rz, which results from the porous nature of coatings produced by the PEO method. Such topography is the result of local plasma discharges, leading to the formation of micropores, cracks and significant height differences on the surface.

The application of an additional polymer coating to the PEO layer resulted in a significant reduction in all analyzed roughness parameters. The reduction in roughness observed in all polymer-coated samples can be attributed to the partial filling and sealing of pores in the PEO layer by the polymer, which reduces the height of surface irregularities and smooths the surface topography.

**Figure 5:**
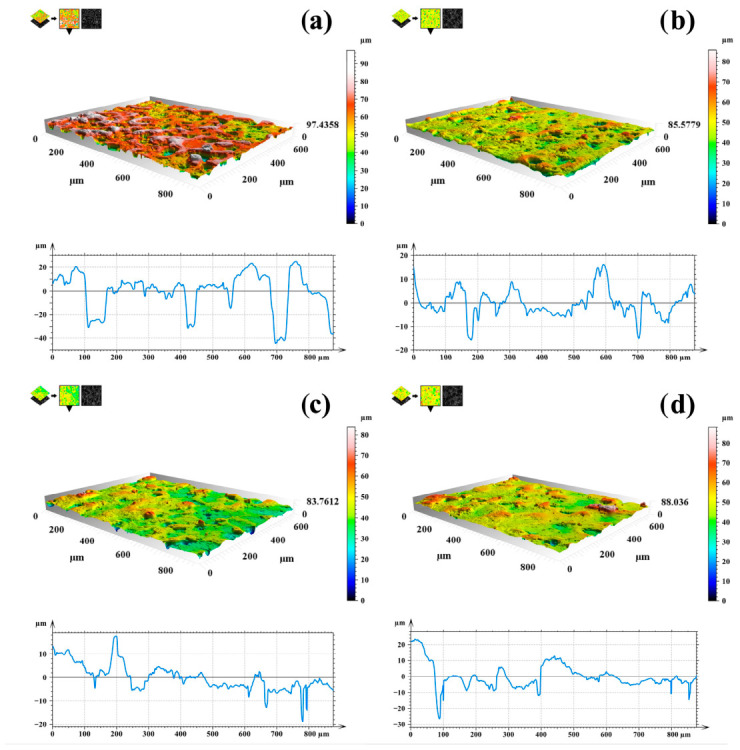
3D topographic maps and roughness profiles of the studied samples: (**a**) PEO sample and polymer-modified samples with: (**b**) 10 layers, (**c**) 20 layers, (**d**) 30 layers.

Importantly, no significant differences were observed between samples coated with 10, 20 and 30 layers of polymer (Figure 5). All variants showed very similar roughness parameter values, indicating that the number of polymer layers in the tested range has no significant effect on the overall surface roughness. Further increasing the number of layers did not lead to significant changes in micro-scale topography, which may indicate that a saturation effect has been achieved, in which subsequent polymer layers mainly overlap the previously formed coating and do not significantly affect the surface geometry. These results confirm that the main effect of the polymer layer is to modify the highly porous surface of PEO, rather than to precisely control roughness through the number of polymer layers applied.

Similar roughness measurement results were obtained by Soleymani et al. [48], who demonstrated a significant difference in surface structure between the MAO coating and the hybrid system with an additional polymer layer. The surface roughness after MAO treatment alone was approximately 11.4 µm, while the use of a PCL/chitosan-based polymer coating reduced the Ra value to approximately 4.7 µm. These results indicate that the polymer layer effectively covers and partially smooths the surface irregularities of MAO by filling micropores and cracks, which may beneficially affect the protective properties of the coating. Similarly, Ghahfarokhi et al. [49] demonstrated that depositing a PDMS layer on a PEO coating reduces surface roughness by sealing pores, resulting in a more compact and homogeneous structure. These observations confirm the general trend towards smoothing the surface of ceramic coatings through the use of polymer layers, highlighting their role in improving the integrity and functionality of hybrid coatings.

The roughness of orthopedic implant surfaces is crucial to their functionality, but its impact is ambiguous and requires a balanced approach. The high surface roughness characteristic of PEO coatings may promote bone cell adhesion by providing a more favorable surface for cell attachment. At the same time, increased roughness can enhance protein adsorption and may also influence corrosion behavior due to a larger effective surface area [50]. The use of a polymer coating reduces these adverse effects by smoothing the surface and creating an additional protective barrier without completely eliminating the microstructure that promotes osseointegration.

### 3.4. Wettability

The surface wettability analysis revealed clear differences between the PEO-coated samples and those additionally modified with the polymer layer. The PEO coating exhibited a very low average water contact angle of 12.25°, indicating a strongly hydrophilic surface. Such high wettability is typical for porous coatings obtained by the PEO method and results from their developed topography [51,52,53]. In the case of diiodomethane, complete spreading of the liquid was observed on the PEO surface (contact angle ≈ 0°), which prevented reliable surface free energy (SFE) determination for this sample.

**Figure 6:**
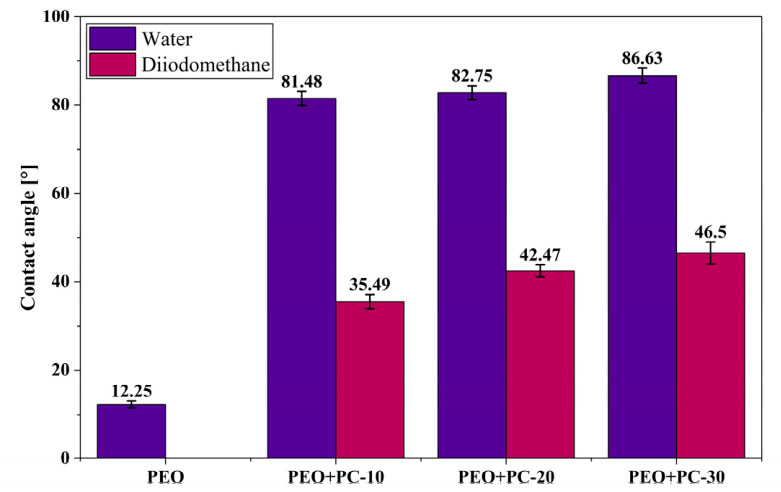
Average contact angle values for the analyzed sample variants.

**Figure 7:**
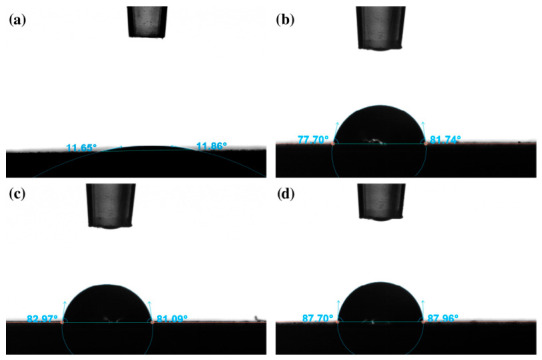
Exemplary photos of water droplets on the surface of samples: (**a**) PEO, (**b**) PEO+PC-10, (**c**) PEO+PC-20, (**d**) PEO+PC-30.

The application of the polymer coating led to a marked increase in the wetting angle to approximately 80–87° (Figure 6 and Figure 7), corresponding to a roughly 7-fold increase compared to the PEO surface. This indicates a change in the chemical nature of the surface, dominated by the properties of the polymer material, and a partial reduction in the influence of the strongly hydrophilic, porous PEO layer. At the same time, all modified samples retained their hydrophilic character (wetting angle < 90°), which is important from the point of view of biomedical applications.

A similar trend was observed for diiodomethane, where the contact angle increased from complete spreading on PEO (0°) to 35.49°, 42.47°, and 46.50° for PEO+PC-10, PEO+PC-20, and PEO+PC-30, respectively. This corresponds to a significant increase in dispersive interaction resistance, confirming the progressive masking of the highly energetic PEO surface by the polymer coating.

The surface free energy (SFE) results further support these observations—Table 4. While SFE could not be determined for the PEO sample due to complete wetting by diiodomethane, a clear decreasing trend was observed for the polymer-coated samples. The total SFE decreased from 43.79 mN/m (PEO+PC-10) to 41.80 mN/m (PEO+PC-20) and 38.35 mN/m (PEO+PC-30), corresponding to an overall reduction of approximately 12.5% between the lowest and highest polymer coverage. This decrease was mainly attributed to the reduction in both dispersive (γ^d^) and polar (γ^p^) components, indicating a progressive dominance of the polymer layer on the surface energetics and a reduction in the high-energy character associated with the PEO coating.

The obtained wettability results are consistent with the literature data on PEO coatings modified with polymer layers. Santos-Coquillat et al. [54] demonstrated that PEO coatings on magnesium are highly hydrophilic, with a wetting angle of approximately 34.1°, while the application of a PCL layer leads to a significant increase in the wetting angle to values close to 83°, indicating a reduction in surface hydrophilicity. A similar trend was observed by Moreno et al. [55], who reported wetting angles of 23.6° for PEO coatings, while after modification with a PLA or PCL layer, these values increased to approximately 95°. The authors attributed this effect to a change in the chemical nature of the surface and the presence of a porous structure in which trapped air can further increase the wetting angle. A similar increase in the wetting angle observed in this study after the application of a polymer layer confirms that the modification of PEO coatings with polymers leads to a transition from a strongly hydrophilic ceramic surface to a surface with moderate wettability, which is typical for PEO/polymer hybrid systems and may be of significant importance from the point of view of their biomedical functionality.

Surface wettability is widely recognized as an important factor influencing biological interactions at the material interface. Surfaces with moderate hydrophilicity have been reported to promote protein adsorption and support the adhesion and activity of osteogenic cells, while potentially limiting bacterial adhesion [56,57,58]. The low contact angle values observed for the PEO coating may lead to increased interaction with physiological fluids, which in the case of magnesium-based materials can contribute to accelerated degradation processes. The application of a polymer coating shifts the wettability toward a more moderate range, which may be beneficial for balancing surface bioactivity and material stability in physiological environments.

### 3.5. Adhesion of Coatings to the Substrate

**Figure 8:**
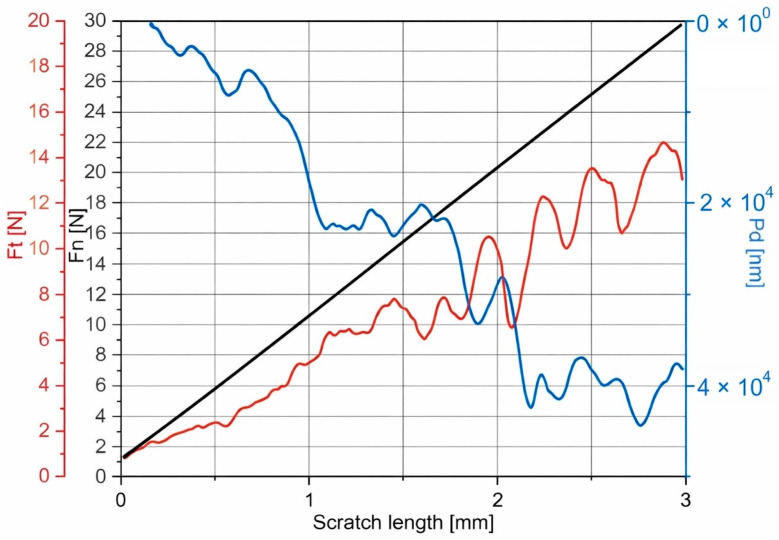
Diagram of the nominal force (black), the frictional force (red) and the penetration depth (blue) as a function of scratch length for PEO+PC-30 sample.

The scratch test results indicate a significant impact of the additional polymer layer on the adhesion of coatings to the substrate (Table 5). For the PEO coating, the lowest critical forces Lc_1_ and Lc_3_ were 1.25 N and 5.45 N, respectively, which indicates limited resistance to damage initiation and complete destruction of the coating. The application of a polymer layer led to a systematic increase in both critical forces, with this effect clearly dependent on the number of polymer layers in the hybrid system. For the PEO+PC-10 and PEO+PC-20 samples, a gradual increase in Lc_1_ to 2.50 N and 3.02 N and Lc_3_ to 8.07 N and 8.48 N, respectively, was observed, indicating improved scratch resistance and better adhesion of the coating to the substrate. The highest critical force values were obtained for the PEO+PC-30 sample, for which Lc_1_ reached 5.07 N and Lc_3_ increased to 9.97 N, confirming a significant improvement in the mechanical integrity of the coating. An example graph from the scratch test is shown in Figure 8. The results obtained suggest that the additional polymer layer effectively increases the adhesion of the coating to the substrate and leads to a delay in both the initiation of damage and the complete destruction of the coating system.

The observed improvement in adhesion can be attributed to the synergistic interaction between the porous PEO layer and the polymer coating. The polymer is able to penetrate and fill micro-pores and cracks within the PEO structure, leading to mechanical interlocking at the PEO/polymer interface and enhanced anchoring of the hybrid system to the substrate [59,60]. In addition, the more compliant and ductile nature of the polymer layer promotes stress redistribution during scratching, reducing stress concentration and delaying crack initiation and propagation compared to the brittle ceramic PEO coating alone. The sealing of structural defects and the formation of a more continuous coating further contribute to improved cohesion and resistance to delamination. Collectively, these effects explain the progressive increase in critical loads with increasing polymer thickness and confirm the beneficial role of the polymer layer in enhancing the overall adhesion and mechanical stability of the hybrid coatings.

### 3.6. Ion Release

**Figure 9:**
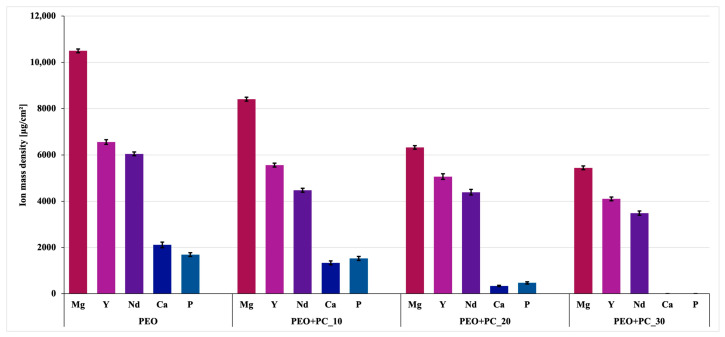
Ion release into the PBS solution during 28 days of incubation at 37 °C for PEO and PEO with 10, 20, and 30 layers of polymer coatings.

The most important aspect of this work was to examine the effect of applying an additional polymer coating, acting as a barrier to slow down the degradation of the modified magnesium alloy. For this purpose, samples after PEO and with different amounts of P(L/G/TMC) polymer layers applied were subjected to an immersion test. Analysis of the ionic composition of the PBS solution after 28 days of sample exposure showed a clear effect of surface modification on the intensity of release of both magnesium alloy elements and PEO layer components (Figure 9). The highest concentrations of Mg, Y, Nd, Ca, and P ions were recorded for the PEO sample, followed by coatings with the lowest number of polymer layers. A quantitative analysis revealed a systematic reduction in ion release with increasing number of polymer layers.

For magnesium ions, the release decreased by approximately 20% for PEO+PC-10, ~40% for PEO+PC-20, and ~48% for PEO+PC-30 relative to the PEO sample, indicating nearly a twofold reduction for the highest number of polymer layers. A similar trend was observed for yttrium and neodymium. For Y, the reduction reached ~15%, ~23%, and ~37%, while for Nd it reached ~26%, ~27%, and ~42% for PEO+PC-10, PEO+PC-20, and PEO+PC-30, respectively. These results indicate a progressive limitation of substrate exposure to the corrosive medium and confirm the increasing barrier effect of the polymer coating.

The most pronounced changes were observed for calcium and phosphorus. Calcium release decreased by approximately 37% for PEO+PC-10 and ~84% for PEO+PC-20, while in the case of PEO+PC-30 no detectable signal was observed. A comparable trend was noted for phosphorus, with a reduction of ~10% for PEO+PC-10 and ~72% for PEO+PC-20, followed by no detectable signal for PEO+PC-30. It should be emphasized that the absence of detectable Ca and P ions for the PEO+PC-30 sample should be interpreted with caution and does not necessarily indicate a complete lack of their release. The observed effect may result from a combination of factors, including a significant reduction in ion transport due to effective pore sealing, possible retention of these ions within the coating structure, and limitations related to the sensitivity of the analytical method. This interpretation is supported by the composition of PEO coatings, which typically contain relatively low amounts of calcium and phosphorus compared to magnesium-based phases [46]. At the same time, the reduction in Ca and P release does not necessarily have to be interpreted negatively, especially if these elements remain bound within the coating structure, where they can potentially participate in osseointegration processes at the implant-tissue interface.

Overall, increasing the number of polymer layers led to a progressive reduction in ion release. These results confirm the enhanced barrier properties of the PEO/polymer system and indicate that increasing coating thickness effectively limits medium penetration and slows degradation-related processes.

It is also worth noting the molar ratio of released calcium and phosphorus ions. Although the Ca:P ratio in the electrolyte used during the PEO process was approximately 1:20, the ratio in the solution after the immersion test was closer to 1:1. Previous XRD studies [46] have shown that the resulting layer contains stable calcium phosphate phases, which are mainly present in the outer part of the coating, which explains the significant release of these elements, especially in the initial phase of incubation. The presence of Ca and P elements is beneficial due to their potential bioactivity and ability to support bone tissue mineralization processes.

The results obtained are consistent with reports in the literature indicating that the application of additional polymer layers on porous PEO coatings effectively limits ion transport to the corrosive environment, acting as a protective barrier for the magnesium substrate [29,30,61,62]. It has been demonstrated that biodegradable polymers penetrate the micropores of the PEO layer, leading to their sealing and a reduction in the release of Mg^2+^ ions and alloying elements, which directly translates into a slowdown in degradation processes. In this study, the sample with 30 polymer layers showed the highest effectiveness in limiting ion transport, which indicates the most developed barrier properties of this system and confirms that increasing the number of layers leads to more effective sealing of the PEO structure. From the point of view of orthopedic applications, controlling the release of magnesium ions and alloying elements is crucial for maintaining the mechanical stability of the implant in the initial period after implantation and for limiting undesirable tissue reactions.

**Figure 10:**
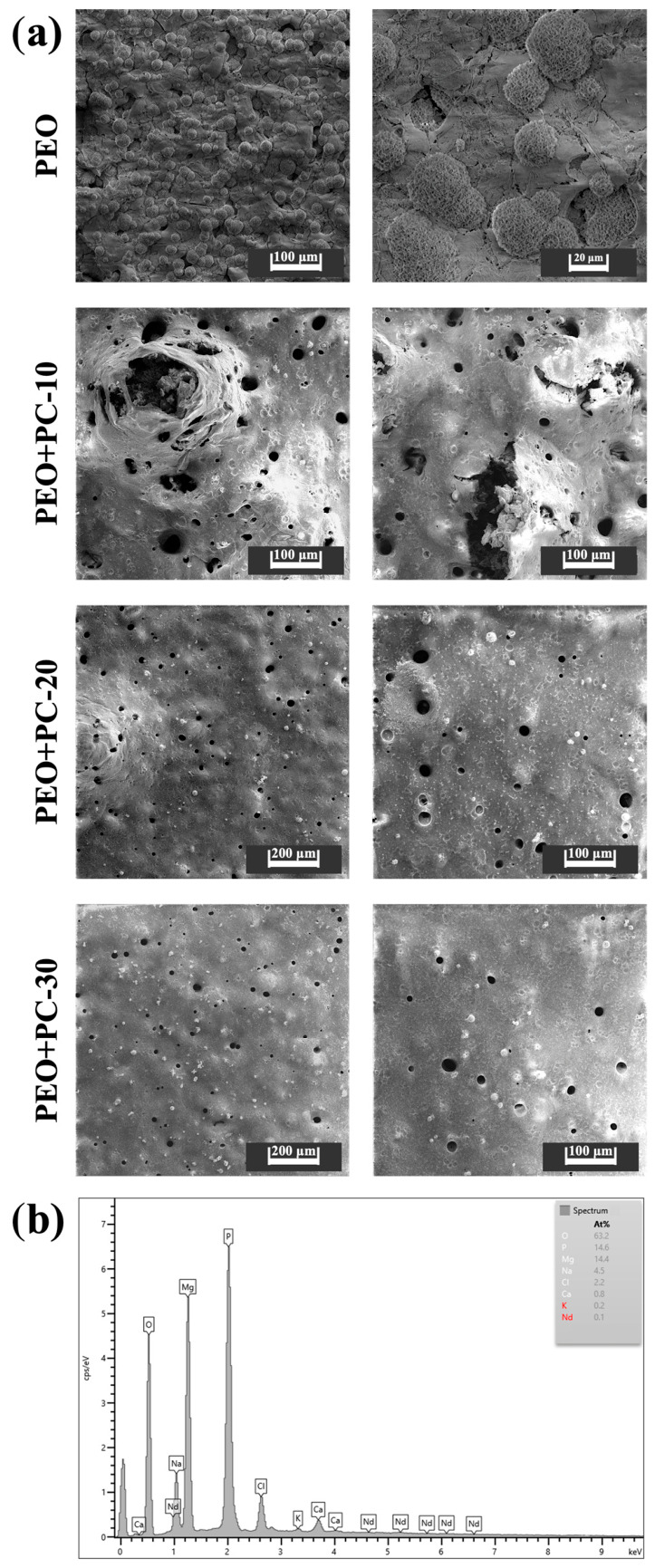
Surface morphology of PEO and PEO with polymer coating samples after 28 days of exposure to PBS solution (**a**) and EDS spectrum of degradation products for PEO sample (**b**).

After completing the immersion tests, the samples were dried and observed using scanning electron microscopy to assess their surface morphology (Figure 10a). Analysis of the SEM images revealed significant differences between the tested variants depending on the number of polymer layers applied. For samples coated only with a layer of PEO, spherical deposits of degradation products are observed, identified in EDS analysis as magnesium oxides and magnesium phosphates (Figure 10b), but it is evident that a layer undergoes delamination and cracking, which indicates its degradation. In the case of the PEO+PC-10 sample, advanced degradation of the coating was observed, manifested by numerous discontinuities, local tears in the polymer layer, and the presence of corrosion products. The characteristic spherical shape of the observed irregularities may indicate intense hydrogen evolution, which confirms the insufficient barrier properties of the coating and allows a significant amount of PBS solution to penetrate the magnesium substrate [63,64,65].

In the case of the PEO+PC-20 variant, local spherical surface irregularities are also visible, which can be associated with sub-coating hydrogen release as a result of solution penetration through the polymer layer. However, the surface morphology does not indicate complete coating rupture, which distinguishes this variant from the PEO+PC-10 sample. The presence of such irregularities indicates that the corrosive medium locally reaches the magnesium substrate, initiating a cathodic reaction, but this process is limited and does not lead to rapid destruction of the coating. The absence of extensive cracks or delamination suggests that the 20 polymer layers provide partial barrier protection that slows down degradation.

By contrast, no significant changes in surface morphology were observed for the PEO+PC-30 sample after 28 days of exposure, compared to the initial state. The surface remained homogeneous with no visible irregularities, cracks or areas of delamination, which indicates an effective reduction in the transport of the PBS solution to the magnesium substrate. This clearly confirms the effective barrier function of the 30-layer polymer coating during long-term exposure.

The results obtained indicate that increasing the thickness of the polymer coating leads to more effective reduction in degradation processes, especially in the initial period of exposure to a corrosive environment, when the rate of degradation of magnesium alloys is highest.

### 3.7. Scanning Acoustic Microscopy (SAM)

**Figure 11:**
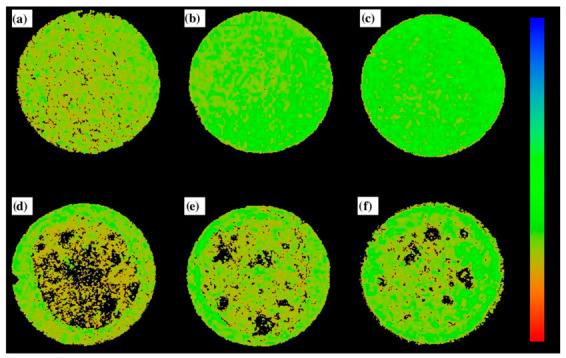
Scanning acoustic microscope images of the surfaces of samples after contact with an aqueous medium. The black areas show hydrogen bubbles released during degradation. Analysis for samples in their initial state: (**a**) 10 layers, (**b**) 20 layers, (**c**) 30 layers and for samples after 28 days of exposure to PBS solution: (**d**) 10 layers, (**e**) 20 layers, (**f**) 30 layers.

Samples with applied polymer coatings in their initial state and after exposure to PBS solution were analyzed using a scanning acoustic microscope. Before the immersion test, the side surfaces and edges of the samples were sealed with tape to ensure that only the polymer-coated surface was exposed to the solution, and hence the most intense corrosion processes are visible in the central part of the samples. Due to the high reactivity of the magnesium substrate in an aqueous environment, local hydrogen release resulting from corrosion processes was observed during the measurements. The formation of gas bubbles locally altered the effective path of the acoustic wave and shifted the focal plane relative to the selected imaging depth. As a result, the reflected echoes originated from planes outside the predefined focal region, leading to the appearance of dark areas in the corresponding SAM images (Figure 11). These regions do not indicate the absence of material or surface defects, but result from a local mismatch between the imaging plane and the actual position of the sample surface caused by the presence of the gas phase at the solid-liquid interface.

Although scanning acoustic microscopy is not a standard method for quantitative assessment of the corrosion resistance of magnesium alloys, the approach used allowed for qualitative visualization of areas with increased corrosion-related activity, manifested by more intense hydrogen evolution. The distribution and intensity of areas of acoustic signal loss were analyzed comparatively for samples with a PEO/polymer hybrid coating. It should be noted, however, that the obtained signal variations may be influenced by multiple factors, such as acoustic mismatch between phases, surface roughness, or imaging artifacts. Nevertheless, the images provide useful information on the uniformity of protection provided by the tested coatings and on the presence of local critical areas where medium penetration and corrosion processes may occur.

Analysis of images obtained using scanning acoustic microscopy revealed significant differences in the behavior of the tested coatings depending on the number of polymer layers applied. Already in the initial state, immediately after immersion in the corrosive medium, numerous areas of hydrogen release initiation were observed for the PEO+PC-10 sample (Figure 11a), located almost over the entire surface. This indicates rapid penetration of the medium through the polymer coating and early initiation of corrosion processes within the magnesium substrate. In the case of samples with 20 (Figure 11b) and 30 (Figure 11c) polymer layers, only a few small areas of such initiation were observed, which indicates more effective barrier properties of thicker coatings already at the initial stage of exposure.

Much clearer differences became apparent after the immersion test. For the PEO+PC-10 sample (Figure 11d), virtually the entire surface was affected by intense hydrogen evolution, confirming progressive and extensive sub-coating degradation. In contrast, for the PEO+PC-20 and PEO+PC-30 samples, this process was limited to local areas of corrosion initiation. A comparison of the two variants shows that the sample with 30 polymer layers had fewer small spots of degradation and several larger ones (Figure 11f), more isolated areas of corrosion activity, while the sample with 20 layers showed both larger areas and a greater number of smaller initiation spots (Figure 11e). The observations confirm that increasing the number of polymer layers leads to a significant reduction in the penetration of the corrosive medium and delays the initiation of the cathodic reaction associated with hydrogen evolution. This trend remains fully consistent with the results obtained from complementary characterization techniques. In the case of the sample coated with 10 polymer layers, extensive hydrogen evolution observed in SAM images corresponded well with the severe surface damage and rupture of the polymer layer revealed by SEM, as well as with the increased release of ions into the solution, indicating insufficient barrier protection. In contrast, samples with thicker polymer coatings exhibited only localized hydrogen evolution, which correlated with minor morphological changes after immersion and markedly lower concentrations of released Ca and P ions. The consistent correlation between acoustic imaging, post-immersion surface morphology, and ion release measurements indicates that the enhanced integrity and continuity of thicker polymer layers effectively limit electrolyte transport, thereby improving the barrier performance of the coating system and slowing down the overall degradation process.

## 4. Conclusions

Based on the conducted research, the following conclusions were drawn regarding the impact of the number of polymer layers on the barrier and degradation-related properties of the tested coating systems:

- The poly(lactide-glycolide-trimethylene carbonate) (P(L/G/TMC)) coating applied by ultrasonic spray coating technique reduced the porosity of the structure by a 4-fold compared to plasma electrolytic oxidation (PEO), as confirmed by scanning electron microscopy (SEM) analyses. Increasing the number of deposition layers resulted in gradual filling of the pores and densification of the coating, while Fourier transform infrared spectroscopy (FT-IR) mapping showed uniform distribution of the polymer across the entire tested surface.
- Compared to PEO alone, the application of the polymer layer altered the surface properties, leading to an approximately 7-fold increased contact angle, 50% reduced surface roughness, and improved coating adhesion. However, no significant differences in these parameters were observed between the polymer-coated samples (10–30 layers). This indicates that the barrier performance is governed primarily by factors such as progressive pore sealing, increased coating continuity, and reduced permeability to the corrosive medium.
- Ion release analysis demonstrated a progressive decrease in the amount of ions migrating into the solution with increasing number of polymer layers, indicating improved barrier performance of the hybrid coatings. In particular, the release of magnesium ions was reduced by approximately 48% for the 30-layer system compared to PEO, with similar trends observed for other alloying elements. The lowest ion release was consistently observed for the 30-layer coating.
- Post-immersion SEM observations indicated improved structural stability of the coatings with increasing number of polymer layers. More pronounced degradation features were observed for thinner coatings, while the 30-layer system showed the highest resistance to morphological changes after immersion.
- Scanning acoustic microscopy (SAM) provided qualitative insight into the distribution of regions associated with degradation processes. However, a decrease in the number and intensity of such regions was observed with an increasing number of polymer layers. These observations should be interpreted cautiously as indirect indicators.

Based on the results obtained, the coating with 30 polymer layers was identified as the most promising configuration in terms of barrier performance and structural stability under the applied experimental conditions. It is emphasized that these conclusions are based on comparative analysis of degradation-related indicators rather than direct quantitative corrosion measurements.

Future work will focus on a comprehensive evaluation of corrosion behavior using quantitative methods, including electrochemical techniques and hydrogen evolution measurements. Additionally, further studies will explore the potential of the developed coating system for functionalization with therapeutic agents.

## Figures and Tables

**Table 1:** Parameters used for applying P(L/G/TMC) polymer coating using ultrasonic spray coating technique.

Deposition Parameters	
Ultrasonic frequency	60 kHz
The power of ultrasounds	1.5 W
Solution flow rate	1 cm^3^/min
Head traverse speed	5 mm/s
Air curtain pressure	2 Pa

**Table 2:** Quantitative analysis of surface morphology parameters (pore density, porosity, and equivalent pore diameter) of PEO and PEO/polymer coatings based on SEM images.

Sample	Pore Density [Pores/mm^2^]	Porosity [%]	Equivalent Diameter [μm]
PEO	210	12.9	28.0
PEO+PC-10	151	4.3	19.0
PEO+PC-20	138	3.7	18.7
PEO+PC-30	122	3.3	18.6

**Table 3:** Mean values with standard deviation of the surface topography parameters determined for the tested samples.

Sample	Ra [μm]	Rz [μm]	Sa [μm]
PEO	11.79 (±1.49)	65.23 (±4.77)	12.29 (±0.97)
PEO+PC-10	5.72 (±0.87)	42.54 (±5.76)	5.84 (±1.45)
PEO+PC-20	4.60 (±1.38)	32.11 (±10.20)	6.20 (±0.37)
PEO+PC-30	5.44 (±1.58)	45.19 (±13.42)	5.60 (±0.21)

**Table 4:** Surface free energy (SFE) and its dispersive (γ^d^) and polar (γ^p^) components for tested samples.

Sample	γ^d^ [mN/m]	γ^p^ [mN/m]	SFE [mN/m]
PEO	-	-	-
PEO+PC-10	40.90	2.90	43.79
PEO+PC-20	39.21	2.59	41.80
PEO+PC-30	36.19	2.17	38.35

**Table 5:** Scratch test results: mean values with standard deviation of the forces Lc_1_—first damage and Lc_3_—complete breakage of the coating.

Sample	Lc_1_ [N]	Lc_3_ [N]
PEO	1.25 (±0.11)	5.45 (±0.28)
PEO+PC-10	2.50 (±0.45)	8.07 (±0.70)
PEO+PC-20	3.02 (±0.51)	8.48 (±0.38)
PEO+PC-30	5.07 (±0.49)	9.97 (±1.42)

## Data Availability

The original contributions presented in this study are included in the article. Further inquiries can be directed to the corresponding authors.
